# Extra renal calyces, precaval renal artery and a variant right testicular artery – are they interrelated? A case report with embryological insight

**DOI:** 10.1590/1677-5449.202401522

**Published:** 2025-08-29

**Authors:** Kamalesh Saravanan, Jayashree Raja, Surabhi AS, Monica Baxla, Seema Singh

**Affiliations:** 1 All India Institute of Medical Sciences – AIIMS, Department of Anatomy, New Delhi, India.

**Keywords:** renal vascular variation, extrarenal calyces, precaval renal artery, accessory renal arteries, variação vascular renal, cálices extrarrenais, artéria renal anterior à veia cava, artérias renais acessórias

## Abstract

Variations in the renal vasculature and testicular artery are common within the human body. However, extra renal calyces and precaval renal arteries are uncommon. In the present case we report extra renal calyces of both kidneys, two right precaval renal arteries, and an abnormal right testicular artery. The kidneys and gonads develop from the intermediate mesoderm. The arteries supplying them bud from the dorsal aorta. This vascular plexus is arranged in a ladder-like manner due to the ascent of kidneys and descent of gonads. Any variation in the vascular supply of these structures must be interrelated, hence care has been taken to clarify the embryological aspect of these variations.

## Resumo

Variações na vascularização renal e na artéria testicular são comuns no corpo humano. No entanto, cálices extrarrenais e artérias renais anteriores à veia cava são incomuns. No presente caso, relatamos a presença de cálices extrarrenais em ambos os rins, duas artérias renais direitas anteriores à veia cava e uma artéria testicular direita aberrante. Os rins e as gônadas se desenvolvem a partir do mesoderma intermediário. As artérias que os irrigam se originam da aorta dorsal. Esse plexo vascular é disposto de forma semelhante a uma escada devido à ascensão dos rins e à descida das gônadas. Qualquer variação no suprimento vascular dessas estruturas deve estar inter-relacionada; por isso, procurou-se esclarecer o aspecto embriológico dessas variações.

## INTRODUCTION

The kidneys are supplied by the right and left renal arteries respectively which are the lateral branches emerging from the abdominal aorta. Accessory arteries are also sometimes present in addition to renal arteries in about 30% of cases.^1^

Anatomical variations involving the renal vasculature are not uncommon and can have significant implications for surgical or radiological procedures. Understanding these variations and how they develop embryologically is crucial to avoid iatrogenic injuries.

Surgical procedures for kidneys such as partial nephrectomy and transplant nephrectomy are frequently performed to treat conditions such as renal tumors, renal trauma, and end stage renal disease.^2,3^ Knowledge of anatomical variations in the renal vasculature is crucial during these procedures to avoid injury to accessory arteries and ensure adequate blood supply to the kidney. Understanding the anatomical relationship between the renal and gonadal vasculature is important during these procedures to avoid damage to gonadal vessels which later can lead to infarction when damaged.

In this case report, we present a combination of anatomical variations in the renal vasculature and associated structures. The cadaver used in the study was procured following institutional guidelines for teaching and research. Hence, the need for clearance from the Ethics Committee was precluded in this study.

## CASE REPORT

**Figure 1:**
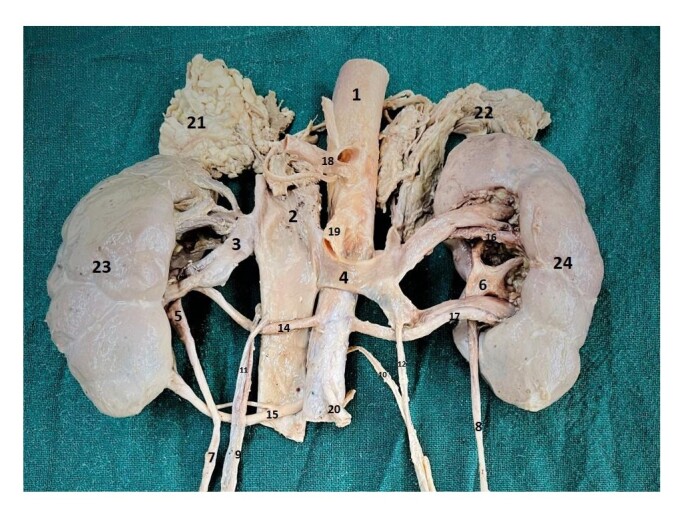
Anterior view of the kidneys and associated structures; 1 – abdominal aorta; 2 – inferior vena cava; 3 – right renal vein; 4 – left renal vein; 5 – right pelvis; 6 – left pelvis; 7 – right ureter; 8 – left ureter; 9 – right testicular artery; 10 – left testicular artery; 11 – right testicular vein; 12 – left testicular vein; 14 – right hilar renal artery; 15 – right lower polar renal artery; 16 – left upper hilar renal artery; 17 – left lower hilar renal artery; 18 – coeliac trunk; 19 – superior mesenteric artery; 20 – inferior mesenteric artery; 21 – right suprarenal gland; 22 – left suprarenal gland; 23 – right kidney; 24 – left kidney.

**Figure 3:**
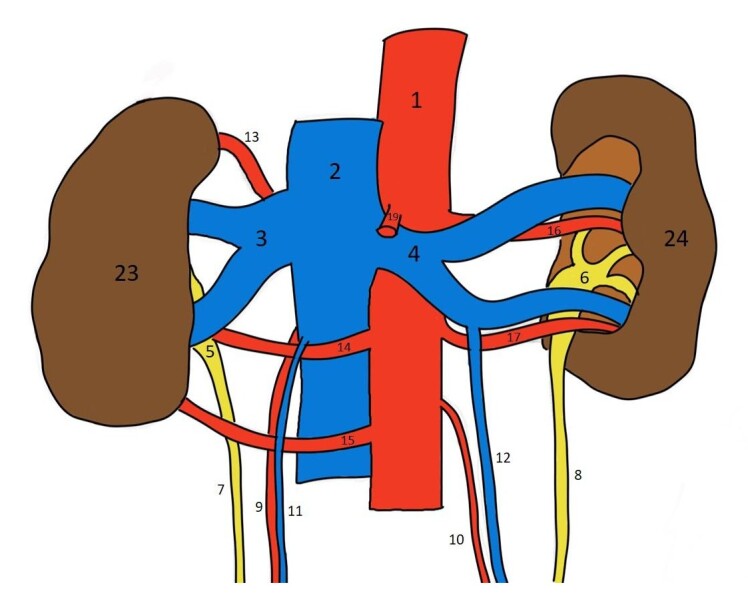
Schematic diagram of the anterior view of the kidneys and associated structures; 1 – abdominal aorta; 2 – inferior vena cava; 3 – right renal vein; 4 – left renal vein; 5 – right pelvis; 6 – left pelvis; 7 – right ureter; 8 – left ureter; 9 – right testicular artery; 10 – left testicular artery; 11 – right testicular vein; 12 – left testicular vein; 13 – right upper polar renal artery; 14 – right hilar renal artery; 15 – right lower polar renal artery; 16 – left upper hilar renal artery; 17 – left lower hilar renal artery; 19 – superior mesenteric artery; 23 – right kidney; 24 – left kidney.

During routine dissection for postgraduates of a 70/M cadaver the following variations were observed. In the right kidney there was an unusual arterial pattern consisting of three distinct renal arteries. These arteries were anatomically arranged as follows: the first artery travelled towards the upper pole (upper polar artery); the second artery towards the hilum (hilar artery), and the third artery coursed towards the lower pole (lower polar artery) (Figures 1 and 3). The hilar artery and the lower polar artery were observed to be precaval in position, that is they travelled in front of the inferior vena cava (IVC) after taking origin from the abdominal aorta (Figures 1 and 3). In contrast, the left kidney featured two arteries, both going towards the hilum (Figures 1 and 3). These arteries were named the upper and lower hilar arteries, based on their positions at the hilum.

**Figure 2:**
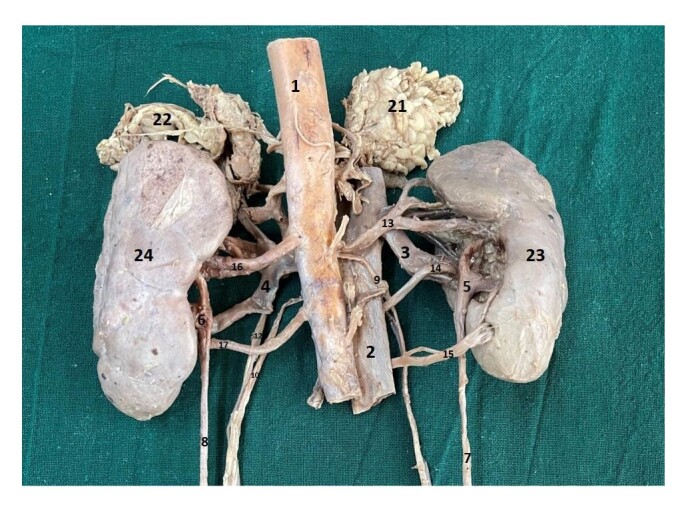
Posterior view of the kidneys and associated structures; 1 – abdominal aorta; 2 – inferior vena cava; 3 – right renal vein; 4 – left renal vein; 5 – right pelvis; 6 – left pelvis; 7 – right ureter; 8 – left ureter; 9 – right testicular artery; 10 – left testicular artery; 11 – right testicular vein; 12 – left testicular vein; 13 – right upper polar renal artery; 14 – right hilar renal artery; 15 – right lower polar renal artery; 16 – left upper hilar renal artery; 17 – left lower hilar renal artery; 21 – right suprarenal gland; 22 – left suprarenal gland; 23 – right kidney; 24 – left kidney.

**Figure 4:**
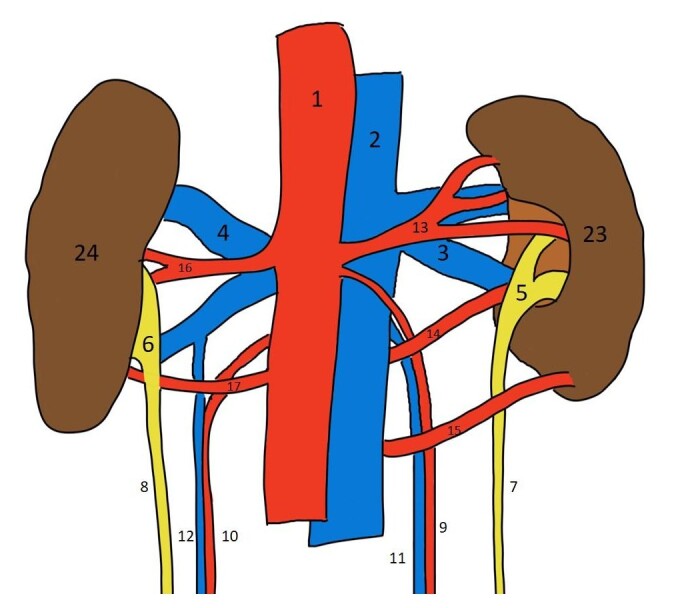
Schematic diagram of the posterior view of the kidneys and associated structures; 1 – abdominal aorta; 2 – inferior vena cava; 3 – right renal vein; 4 – left renal vein; 5 – right pelvis; 6 – left pelvis; 7 – right ureter; 8 – left ureter; 9 – right testicular artery; 10 – left testicular artery; 11 – right testicular vein; 12 – left testicular vein; 13 – right upper polar renal artery; 14 – right hilar renal artery; 15 – right lower polar renal artery; 16 – left upper hilar renal artery; 17 – left lower hilar renal artery; 23 – right kidney; 24 – left kidney.

Most of the time, the origin of the testicular arteries is from the anterolateral aspect of the aorta, just below the origin of the renal artery, and then they descend downwards. In the present case, the right testicular artery was observed to arise just below the right upper polar artery; descending first behind the IVC and then in front of the right hilar and the right lower polar arteries (Figures 1, 2, 3 and 4).

Another unusual feature observed in both kidneys was that the renal pelvis divided into calyces outside the renal parenchyma, a condition referred to as extrarenal calyces. Although the individual calyces are not separately labelled in the illustrations, their presence can be inferred from the region just above the labelled renal pelvis. Additionally, the outer surface of both kidneys appeared lobulated. The extrarenal calyx on the left side is clearly visualized in Figures 1 and 3, while that on the right side is evident in Figures 2 and 4.

## DISCUSSION

Comprehending anatomical variations in the renal vasculature and associated structures is crucial to avoid iatrogenic injuries during invasive procedures involving the kidneys and the adjacent structures. During certain procedures like nephrectomy; kidneys are approached from the posterior aspect.^4^ The presence of precaval renal arteries poses a significant risk of damage if the operating surgeon is unaware of such anomalies. In 2011, Bouali reported around 9% prevalence of this variation.^5^ In cases of leiomyosarcoma involving the IVC, which is a relatively rare condition, surgical resection of the affected segment may be necessary.^6^ The presence of a precaval renal artery adds complexity to such surgical procedure. Therefore, meticulous preoperative planning and intraoperative care are essential to safely manage this anatomical variation and ensure optimal surgical outcomes. The importance of avoiding injury to accessory renal arteries extends beyond preventing blood loss and maintaining a clear surgical field. These accessory renal vessels are the end arteries^7^ and when damaged impair blood flow to the organ, leading to ischemia and infarction.

The course of the testicular arteries is crucial for abdominal and pelvic surgeries because of their anatomical proximity to several key structures, hence knowledge of variations in their course is mandatory to avoid complications such as inadvertent injury, ischemia, or excessive bleeding.^8,9^ In the present case, the right gonadal artery travelled in front of the precaval renal arteries.

The presence of extrarenal calyces is a rare anatomical variation which can sometimes be mistaken for hydronephrosis or renal or perinephric cysts on imaging studies. They can also predispose individuals to formation of renal stones, as they can lead to renal stasis and impaired drainage.^10^

**Figure 5:**
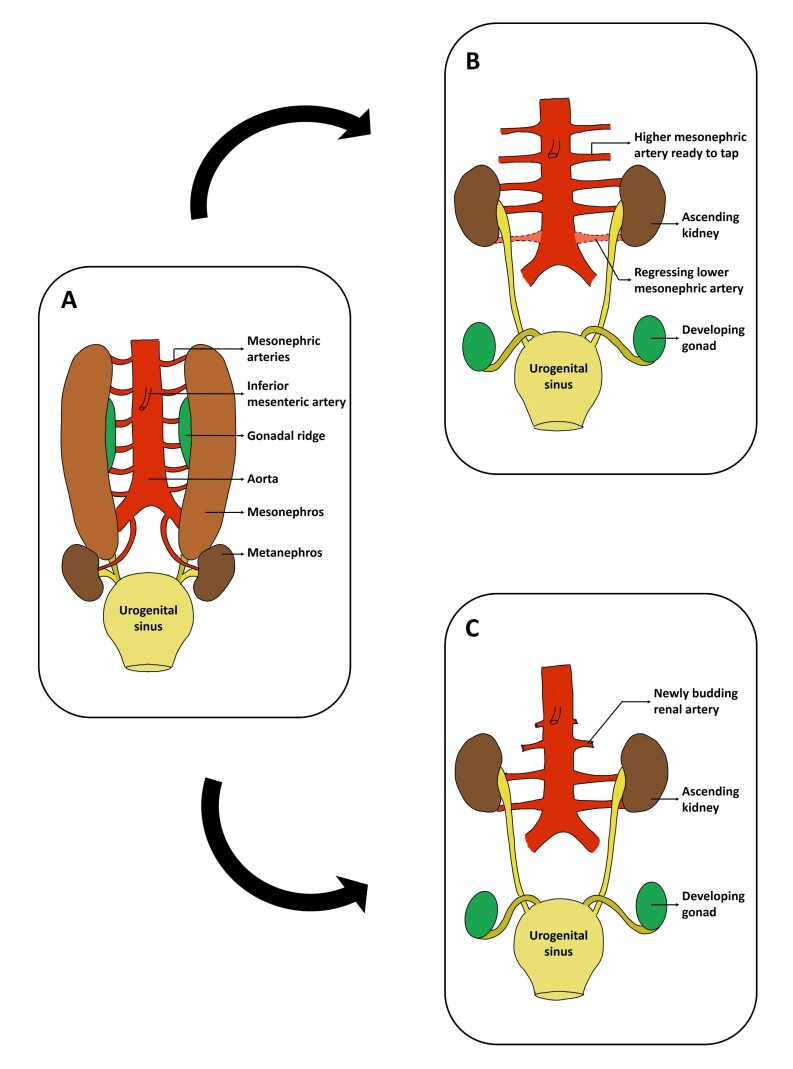
Schematic representation of the embryological development of the kidneys and renal arteries, illustrating two theories of renal vascular development. (A) Developing kidneys, gonads and associated structures; (B) Represents Felix’s classical “step ladder” model of renal artery development, where the renal arteries arise from the mesonephric arteries. As the kidneys ascend, the lower mesonephric arteries regress, and higher ones establish connections to supply the kidneys; (C) Illustrates the newer theory, which challenges the step ladder model. According to recent findings, the mesonephric arteries do not make direct contact with the kidneys. Rather, a vascular capillary plexus emerges de novo from the lateral wall of the abdominal aorta, forming a caudal-to-cranial pattern of renal vascularization as the kidneys ascend.

Embryological aspects: the kidneys as well as the gonads develop from the intermediate mesoderm. The embryonic metanephros gives rise to the adult kidney, which ascends from its initial sacral location to its final lumbar position.^11^ As the developing kidney ascends; the vessels supplying it also start developing from the dorsal aorta in a step ladder manner.^12,13^ As the kidneys ascend, the lower vessels degenerate and the higher ones gradually take over the blood supply^14^ (Figure 5). A recent study proposes that the mesonephric arteries do not establish direct contact with the metanephros. Rather, a vascular bud was observed arising from the lateral wall of the aorta and extending toward the metanephros. This phenomenon may indicate a process of neoangiogenesis, playing a crucial role in development of the definitive renal artery^15^ (Figure 5).

Factors like Vascular Endothelial Growth Factor (VEGF) and its receptors, and also Fibroblast Growth Factor (FGF), are important in angiogenesis and vascular remodelling.^16^ Disruption of these factors could lead to formation of these variations. These accessory arteries can have significant clinical implications during surgical procedures, particularly those involving the kidneys.

The gonads also develop within the intermediate mesoderm from the gonadal ridges present on its medial aspect. Factors like SRY and SOX9 are important in the descent of the testes. The SRY gene triggers a cascade of gene interactions, including SOX9, which is essential for the development of the testes and their descent.^17^ The testes also derive their blood supply from the splanchnic plexus, which includes the ladder vessels associated with renal arteries. Therefore, variations in the renal vasculature can also give rise to variations in the testicular vessels.

The metanephric blastema and the collecting ducts develop by communicating with each other via certain factors like WNT, GDNF, and others.^18^ The presence of extrarenal calyces may be attributed to the fact that either the metanephric blastema developed later or the collecting system developed late.^19^

In conclusion, understanding the intricate embryological development of the urogenital system can give us an idea of all of the variations such as precaval renal arteries, abnormal testicular arteries, and extrarenal calyces. Knowledge of these variations is vital to avoiding complications during surgical interventions and improving patient outcomes.

## Data Availability

All data generated or analyzed during this case report are included in this submitted article. Additional information is available from the corresponding author on reasonable request.
